# Surgical management of an extensive nasal mass in an adolescent: insights from diagnostic imaging and histopathology

**DOI:** 10.1093/jscr/rjae831

**Published:** 2025-01-07

**Authors:** Sebastian Asteguieta, Carlos Diaz, Javier Alarcon, Vanessa Godinez

**Affiliations:** Department of Research, Universidad Francisco Marroquín, 13 av, Guatemala City 01011, Guatemala; Department of Research, Universidad Francisco Marroquín, 13 av, Guatemala City 01011, Guatemala; Department of Research, Universidad Francisco Marroquín, 13 av, Guatemala City 01011, Guatemala; Department of Research, Universidad Francisco Marroquín, 13 av, Guatemala City 01011, Guatemala

**Keywords:** nasal mass, adolescent patients, ocular proptosis, exophthalmos, differential diagnosis

## Abstract

A 17-year-old female presented with a mass in the right nasal fossa and eye protrusion. Imaging revealed a large osseous mass originating from the right turbinates, causing exophthalmos without tissue invasion. A partial resection via the Caldwell–Luc approach was performed, but hemodynamic instability halted the procedure, leaving a residual mass. Histopathology confirmed an osseous lesion with osteoblasts in a hypocellular stroma. The patient required blood transfusions postoperatively due to significant blood loss, but recovered well with antibiotics and supplements and was discharged after mild nausea was managed.

## Introduction

Juvenile ossifying fibroma (JOF) is an uncommon benign fibro-osseous tumor that primarily affects children and adolescents. It tends to occur in the sinonasal region and is characterized by distinct features compared to conventional ossifying fibromas. These include its early onset, predilection for specific anatomical locations, aggressive local growth, and significant risk of recurrence even after surgical resection [1].

Nasal masses in adolescent patients are a rare pathology that can present with a variety of symptoms depending on the size, location, and impact on surrounding structures. The presence of a nasal mass accompanied by ocular proptosis requires a differential diagnosis ranging from benign lesions to malignant neoplasms. This case describes a 17-year-old female patient referred to an otolaryngology center due to the presence of a mass in the right nasal fossa and ipsilateral eye protrusion. Clinical examination and contrast-enhanced CT findings are detailed, emphasizing the key role of imaging in the evaluation and management of such conditions [2].

## Case presentation

A 17-year-old female presented with a mass in the right nasal fossa and ipsilateral eye protrusion. Ocular examination revealed forward displacement of the eyeball with mild restriction in lateral gaze, though visual acuity remained preserved. Rinne and Weber tests were negative, indicating no auditory impairments. No abnormalities were found in the oral cavity, larynx, or neck, and nasal inspection showed an occlusive mass in the right nasal fossa without signs of hemorrhage. Overall, the remainder of the examination was unremarkable.

### Laboratories/imaging findings

**Figure 1:**
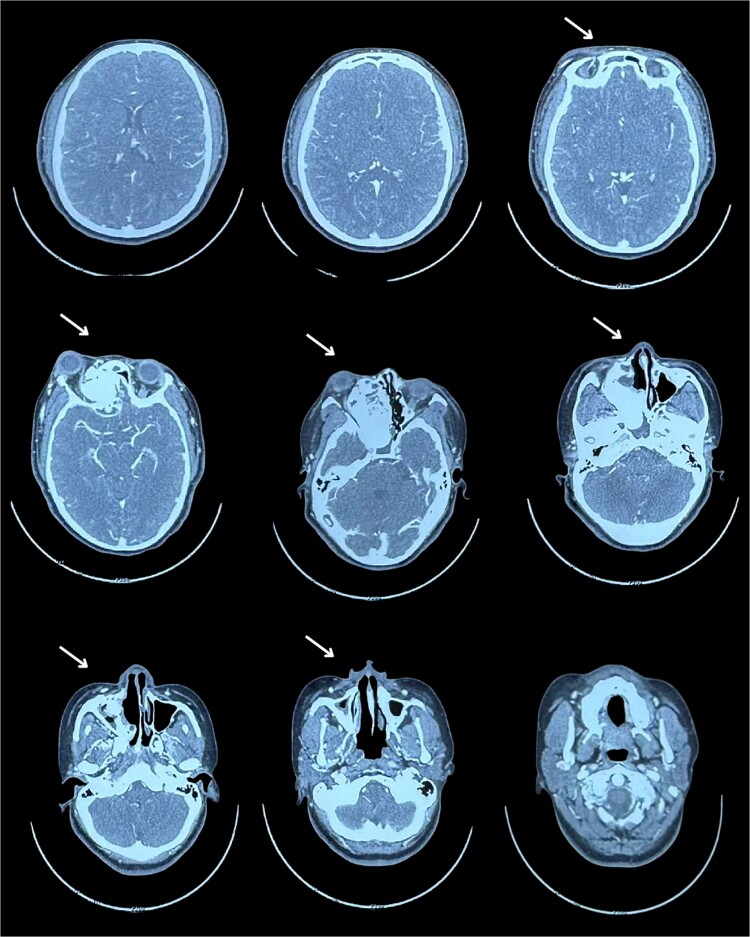
Ossifying fibroma lateral right eye displacement.

A tomography was performed to assess the extent of the mass inside the right nasal cavity. The imaging findings (Fig. 1) were consistent with a large mass (65 × 44 + 39 mm) originating from the right turbinates that was heavily remodeling the medial wall and the floor of the right orbit, compromising internal aspects of the maxillary and ethmoid bones; leading to a superior lateralization of the right eye causing exophthalmos.

Muscular and neural tissue were conserved intact but displaced.

### Surgical intervention

**Figure 2:**
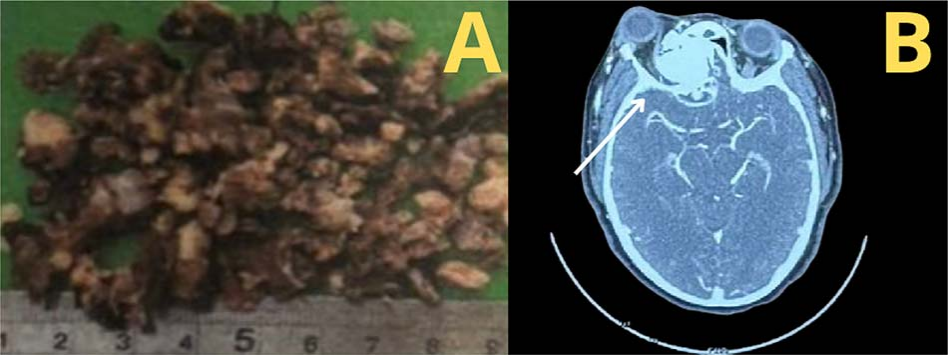
(A) Macroscopic examination of ossifying fibroma, (B) CT.

According to the information gathered, the surgeon decided to perform a partial resection of the maxillary neoplasia (Fig. 2). The chosen approach upon evaluation was the Caldwell–Luc procedure, gaining access to the maxillary sinus. Dimensions and anatomic relationships of the mass were established, determining an extension up to the sphenoid bone covering the ipsilateral choana. A predominantly osseous tumor was observed, with a significant contribution of trabecular bone, vascularized, exhibiting a tendency to hemorrhage. Partial resection of the tumor was accomplished, leading to visualization of the peripheral orbit but not decompressing it totally due to hemodynamic instability during intervention. A residual mass was left at the sphenoidal level and cranial base, a future reintervention was considered.

### Postoperative outcomes and pathology report

After surgery, the patient was transferred to recovery and received packed red blood cell transfusions due to significant blood loss and anemia. She recovered well with mild nausea, stable vital signs, and was discharged with amoxicillin-clavulanate and iron/folate supplements.

The patient's ocular outcome was favorable, with preserved vision and mild lateral gaze restriction, thanks to the surgical team's focus on preserving orbital structures. This was key to her positive recovery. The diagnosis was JOF, a rare benign tumor common in adolescents, which causes aggressive local growth and carries a recurrence risk. Imaging played a critical role in diagnosis and management.

A second intervention was performed with the Caldwell–Luc approach for complete tumor removal.

## Discussion

This case discusses the challenges of managing large nasal masses in adolescents. Despite partial resection, the patient had an uneventful recovery, underscoring the need for a multidisciplinary approach and long-term follow-up. The surgical team opted for the Caldwell–Luc approach due to its effectiveness in providing wide access to the maxillary sinus and adjacent anatomical structures, which was crucial for addressing the large osseous mass. This approach allowed precise visualization and partial resection of the tumor, particularly given its extension into the sphenoid bone and involvement of the ipsilateral choana. Additionally, the approach facilitated the careful management of the mass's vascularity, minimizing further risks of hemorrhage while preserving critical surrounding structures such as the orbital contents [3].

The surgical team opted for the Caldwell–Luc procedure over an extended endoscopic prelacrimal medial maxillectomy (EPMM) due to its proven effectiveness with osseous lesions and the surgeon's familiarity with it. While EPMM offers better visualization and vascular control, the Caldwell–Luc approach was chosen to address the lesion's vascularity and intraoperative conditions. However, its limited access to the sphenoid and choana suggests EPMM may be considered in future cases [4].

The contrast-enhanced CT images revealed that the lesion was highly vascular, consistent with the characteristics of sinonasal ossifying fibromas (OF). This vascularity was indeed considered pre-operatively, as the surgical team anticipated potential challenges in controlling bleeding during resection. However, pre-operative embolization was not pursued in this case. While embolization is commonly used in highly vascular tumors like juvenile nasopharyngeal angiofibroma to reduce blood loss, it was not considered necessary here due to the specific location and nature of the lesion [5].

The patient experienced hemodynamic instability during surgery due to significant blood loss from the highly vascular lesion. Challenges in controlling bleeding, particularly in the suborbital neurovascular bundle and potential injury to the infraorbital maxillary vessels, contributed to the instability, postoperative course was largely uneventful, with recovery following blood transfusions and management of mild nausea. Discharge with antibiotics and supplementation was appropriate due to the risk of infection and anemia. Monitoring of the residual mass at the cranial base is crucial, with future surgical reintervention planned to achieve complete resection if needed [6].

## Conclusion

This case highlights the challenges of managing large nasal masses in adolescents, especially when causing exophthalmos. Diagnosis of an osseous tumor originating from the right turbinates was made through clinical evaluation, imaging, and histopathology. The Caldwell–Luc procedure facilitated partial tumor resection, but residual mass remained due to hemodynamic instability. The patient had a smooth postoperative recovery with medical management. This case emphasizes the importance of a multidisciplinary approach, patient safety, and long-term follow-up to optimize outcomes and reduce recurrence.
